# Exploration of Risk Factors for Poor Prognosis of Non-Traumatic Non-Aneurysmal Subarachnoid Hemorrhage

**DOI:** 10.3390/biom12070948

**Published:** 2022-07-06

**Authors:** Yuan Yuan, Jingjiong Chen, Yaxuan Zhang, Fei Zhao, Yanyu Zhai, Xiaofeng Xu, Lixia Xue, Yuwu Zhao, Hongmei Wang

**Affiliations:** Department of Neurology, Shanghai Jiao Tong University Affiliated Sixth People’s Hospital, Shanghai 200233, China; yybzcmu@163.com (Y.Y.); jjiong76@yeah.net (J.C.); 17860624912@163.com (Y.Z.); zf8642@126.com (F.Z.); 20141490@stu.nun.edu.cn (Y.Z.); xuxiaofeng987620@126.com (X.X.); dr.xue@126.com (L.X.)

**Keywords:** SAH, prognostic, age, neuron-specific enolase

## Abstract

Background: Subarachnoid hemorrhage (SAH) is a devastating neurological disease associated with high rates of mortality and disability. Aneurysms are the main cause of non-traumatic subarachnoid hemorrhages. However, non-traumatic non-aneurysmal subarachnoid hemorrhage (naSAH), another clinical type of SAH, has been poorly studied for its prognosis and risk factors. Method and result: We collected demographic and clinical variables for 126 naSAH and 89 aneurysmal subarachnoid hemorrhage (aSAH) patients, including age and gender; hospitalization days; hematological indicators; clinical score scales; past medical history; and personal history. We found that the monocytes in naSAH (0.50 ± 0.26) patients were lower than in aSAH patients (0.60 ± 0.27). The prevalence of diabetes in naSAH (30.2%) patients was higher than in aSAH (14.5%) patients. The naSAH patients were divided into good and poor outcome groups based on the modified Rankin Scale at the 90th day (90-day mRS) after discharge. A univariate analysis showed that there were significant differences in age, white blood cell count (WBC), monocyte count, D-dipolymer, neuron-specific enolase (NSE), random blood glucose (RBG), aspartate transaminase (AST), urea and free triiodothyronine (FT3) between the two groups. A logistic regression showed that aging and high level NSE were independent risk factors for a poor outcome. The predictive ability of age (area under curve (AUC) = 0.71) and NSE (AUC = 0.68) were analyzed by a receiver operating characteristic (ROC) curve. The results of the logistic regression suggested that age, D-dipolymer, NSE, RBG, urea and FT3 distinguished and predicted the prognosis of naSAH. The discriminant analysis of the above variables revealed that the discriminant accuracy was 80.20%. Conclusions: Compared with aSAHs, naSAHs are more likely to occur in patients with diabetes, and the level of monocytes is lower. Moreover, the prognosis of elderly patients with an naSAH is relatively poor, and the level of NSE in the course of the disease also reflects the prognosis. Multivariate comprehensive analysis is helpful to judge the prognosis of patients at a small cost.

## 1. Introduction

Subarachnoid hemorrhage (SAH) is associated with high morbidity and mortality, with a case fatality rate reaching 25–50% [1]. Non-traumatic non-aneurysmal subarachnoid hemorrhage (naSAH), also known as angiogram-negative subarachnoid hemorrhage, is generally considered a clinical type with a better prognosis than aneurysmal subarachnoid hemorrhage [2]. However, some patients with an naSAH still develop clinical complications or achieve poor functional outcomes despite their mild condition at admission [3]. For example, cognitive deficits have been demonstrated in some patients with an naSAH, which may include deficits in memory, attention and executive function [4].

Previous studies have reported that the modified frailty index [5,6] and stress-induced hyperglycemia [3] are independent predictors of a poor outcome following an naSAH. A recent large-scale clinical retrospective study [7] developed a nomogram to predict the clinical outcomes of patients with an naSAH, and the result showed that a symptomatic vasospasm, high World Federation of Neurological Surgeons (WFNS) grading, cerebral edema, and non-perimesencephalic naSAH after a hemorrhage were associated with a poor outcome of an naSAH. We have not found authoritative studies that have explored the predictions of the prognosis of an naSAH through hematological indicators.

Although there are few studies on the prognostic factors of an naSAH, patients with a poor prognosis should receive extensive attention in clinical practice. To identify independent risk factors for the poor prognosis of an naSAH and explore its prognostic model, a large number of hematological indicators and other variables were collected in the current study.

## 2. Materials and Methods

### 2.1. Patients

**Figure 1:**
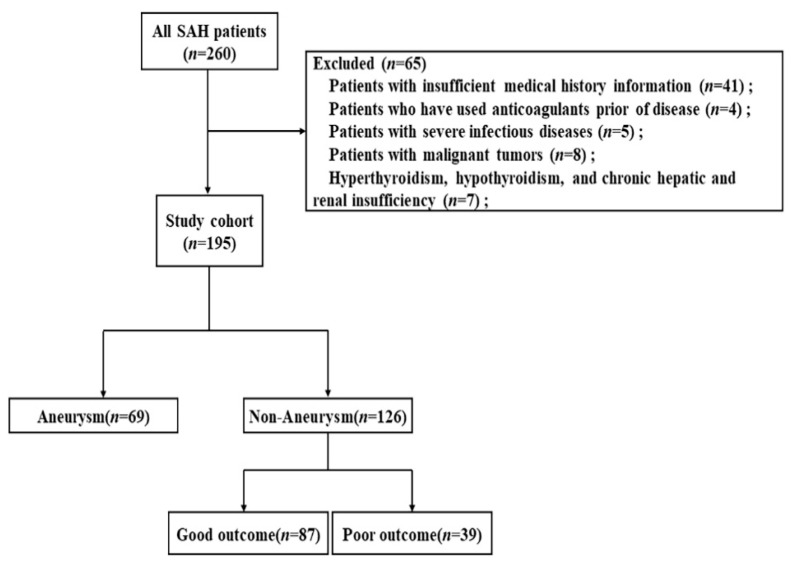
Flowchart of patients included in this study.

We retrospectively collected demographic and clinical variables of 260 patients with naSAH that were admitted to the Neuroscience Intensive Care Unit at the Shanghai Jiao Tong University Affiliated Sixth People’s Hospital (Shanghai, China) from February 2013 to March 2021. SAH was confirmed by head computerized tomography (CT). Non-traumatic SAH without confirmed bleeding source in cerebral digital subtraction angiography (DSA) examination within 72 h of admission was identified as naSAH [3]. Neuron-specific enolase (NSE), which was measured by enzyme-linked immunosorbent assay (ELISA), was a routine test item for inpatients. Inclusion criteria were age >18 years and admitted to hospital within 7 days of onset. Exclusion criteria were patients with insufficient medical history information (41); patients who had used anticoagulants prior to disease (4); patients with severe infectious diseases (5); patients with malignant tumors (8); patients with hyperthyroidism, hypothyroidism, and chronic hepatic and renal insufficiency (7). Total of 195 cases were included in the study eventually. These patients were divided into aneurysmal subarachnoid hemorrhage (aSAH) (69) and naSAH (126) groups, and the naSAH patients were then divided into good (87) and poor (39) outcome groups based on the 90-day mRS (Figure 1). This study was approved by the ethics committee of Shanghai Jiao Tong University Affiliated Sixth People’s Hospital in Shanghai, China.

### 2.2. Clinical Factors

Baseline characteristics included age, gender, past history of SAH and cerebral infarction, personal history (history of smoking and drinking) and presence of co-morbidities (high blood pressure (HBP) and diabetes mellitus (DM)). The conditions at admission were determined by Glasgow Coma Scale (GCS): GCS 15 was defined as conscious, GCS 13~15 as mild coma, GCS 9~12 as moderate coma and GCS 3~8 as severe coma.

Post-admission data included (1) hospitalization days; (2) blood leukocyte level, blood coagulation examination, NSE, blood glucose and lipid; (3) liver, kidney and thyroid function examination; (4) incidence of pneumonia, and modified Fisher Scale (mFS).

Clinical outcome at three months was determined by 90-day mRS. mRS 0–2 was defined as good outcomes and mRS 3–6 as poor outcomes.

### 2.3. Statistical Analyses

First, baseline characteristics and post-admission data of aSAH and naSAH were compared. The naSAH patients were divided into poor outcome (mRS 3–6) and good outcome (mRS 0–2) groups based on 90-day mRS. Then univariate analyses were conducted. Variables with a *p*-value < 0.05 in univariate analysis were included in the binary logistic regression model to identify the independent risk factors of poor outcomes. Meanwhile, odds ratio (OR) and 95% confidence interval (Cl) were calculated by logistic regression. The independent risk factors were performed with receiver operating characteristic (ROC) curve analysis and the area under curve (AUC) was calculated to assess the ability of the factors to predict poor outcomes.

In addition to screening independent factors, the results of logistic regression analysis showed the factors that can distinguish and predict the clinical outcome. These factors were incorporated into discriminant analysis to establish a discriminant model. Subsequently, 126 naSAH patients were grouped according to the cut-off value of age determined by the ROC curve and correlation analysis performed separately.

Moreover, the proportion–proportion plots were performed to assess data distribution. Normally and non-normally distributed variables were expressed as means ± standard deviations and median (interquartile range), respectively. Nominal or categorical variables were expressed as the number of patients (percentage). Student’s t-test was used to compare the normally distributed variables while Mann–Whitney U test was performed to compare non-normally distributed variables. Chi-square, likelihood-ratio chi-square or Cochran–Armitage trend tests were used to compare the categorical variables as appropriate. For correlation analyses, variables were evaluated by Spearman’s rank correlation coefficient. Statistical analysis was performed using SPSS statistical software. *p*-value < 0.05 was defined as statistically significant.

## 3. Result

### 3.1. Clinical Data

Details of the aSAH patients are summarized in Table 1 and compared with the naSAH patients. Table 2 shows the baseline characteristics, post-admission data and outcomes of the patients with an naSAH. There were 59 naSAH and 23 aSAH patients who were conscious (admission-GCS: 15), 24 naSAH and 10 aSAH patients with mild coma (admission-GCS: 13~14), 11 naSAH and 15 aSAH patients with moderate (admission-GCS: 9~12) coma and 32 naSAH and 21 aSAH patients with severe coma (admission-GCS: 3~8). In addition, the modified Fisher scale was collected. Fifty-one naSAH and 17 aSAH patients had only a small amount of blood (mFS: 1), 18 naSAH and 16 aSAH patients had a small amount of blood with an intraventricular hemorrhage (mFS: 2), 13 naSAH and 9 aSAH patients had a massive hemorrhage (mFS: 3), and 44 naSAH and 27 aSAH patients had a massive amount of blood with an intraventricular hemorrhage (mFS: 4).

### 3.2. Comparison between Patients with aSAH and naSAH

**Figure 2:**
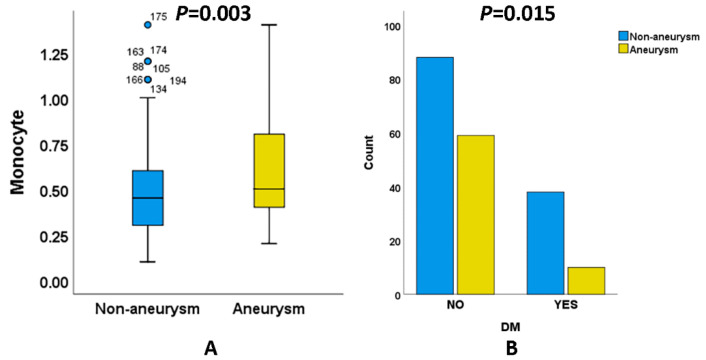
Clinical indicators of significant difference between aneurysmal subarachnoid hemorrhage and non-aneurysmal subarachnoid hemorrhage. (**A**) Absolute count of monocytes in blood. (**B**) The number of diabetic patients.

Because patients with aSAH and naSAH received different treatment methods, this study focused on the clinical factors during the period from admission to treatment. We found that the absolute count of monocytes (Figure 2A) in naSAH (0.50 ± 0.26) patients was lower than in aSAH (0.60 ± 0.27) patients. In addition, the proportion of diabetes (Figure 2B) in naSAH (30.2%) was higher than in aSAH (14.5%), suggesting that metabolic disorder may promote the occurrence and development of an naSAH. However, there was no significant change in the RBG levels between the two groups.

### 3.3. Differences in Age and Hematological Indicators between Good Outcome and Poor Outcome of naSAH Patients

**Figure 3:**
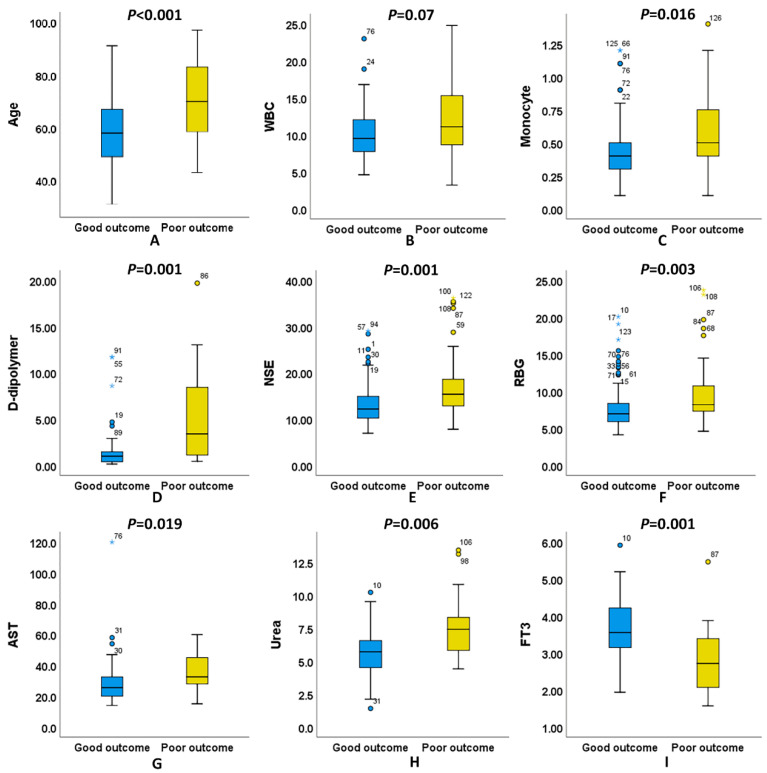
There were significant differences in several clinical indicators between poor and good outcome of naSAH. (**A**) Age. (**B**) WBC. (**C**) Monocyte. (**D**) D-dipolymer. (**E**) NSE. (**F**) RBG. (**G**)AST. (**H**) Urea. (**I**) FT3.

The average age (Figure 3A) was higher in the poor outcome group (70.03 ± 15.17) than in the good outcome group (58.45 ± 12.26), indicating that the clinical outcomes of elderly patients tended to deteriorate. In addition, some hematological indicators with significant differences between the two groups were found in this study. As shown in Table 2, WBC (Figure 3B), absolute count of monocytes (Figure 3C), D-dipolymer (Figure 3D), NSE (Figure 3E), RBG (Figure 3F), AST (Figure 3G) and urea (Figure 3H) levels in the blood of patients with a poor outcome were significantly higher than those of patients with a good outcome, while the FT3 (Figure 3I) level was lower than in patients with a good outcome.

### 3.4. Comparison of Pneumonia Incidence, Clinical Scale, and Previous Disease History in Patients with naSAH

**Figure 4:**
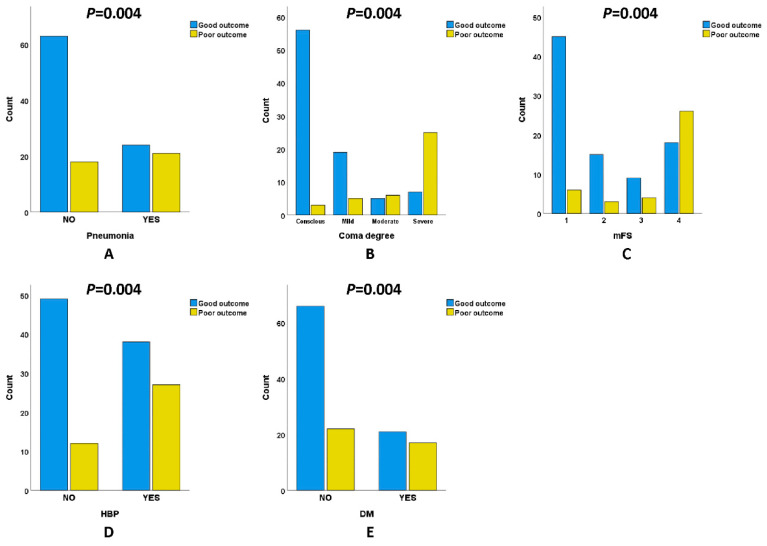
Compared with the good outcome group, patients with a poor outcome had a worse course of disease and past history. (**A**) Incidence of pneumonia. (**B**) Degree of coma at admission. (**C**) The severity of the bleeding. Proportion of people with HBP (**D**) and DM (**E**).

We next observed the relationship between clinical outcomes and the occurrence of pneumonia, GCS, mFS and previous disease history in patients with an naSAH. Whether or not pneumonia occurred during the course of the disease reflected the systemic immune level and inflammatory state of the patients. Table 2 shows that the proportion of pneumonia (Figure 4A) that occurred in the poor outcome group (53.8%) was higher than in the good outcome group (27.6%). GCS is a scale to evaluate the degree of coma at admission. As shown in Figure 4B, the degree of coma at the admission of patients with a poor outcome (GCS 3~8: 64.1%) was higher than patients with a good outcome (GCS 3~8: 8.0%). The mFS is another scale based on CT to assess the severity of an SAH. Similar to GCS, the proportion of the poor outcome group (mFS 1: 15.4%; 2: 7.7%; 3: 10.3%; 4: 66.7%) increased gradually with the severity of hemorrhage, while the proportion of the good prognosis group (mFS 1: 51.7%; 2: 17.2%; 3: 10.3%; 4: 20.7%) showed the opposite (Figure 4C). Subsequently, previous disease history including HBP and DM was investigated. Table 2 shows that the prevalence of HBP (Figure 4D) and DM (Figure 4E) in the poor outcome group (HBP:69.2%; DM:43.6%) was higher than in the good outcome group (HBP:43.7%; DM:24.1%).

### 3.5. Screening of Independent Predictors of Adverse Prognosis of naSAH and Evaluation of Their Predictive Ability

**Figure 5:**
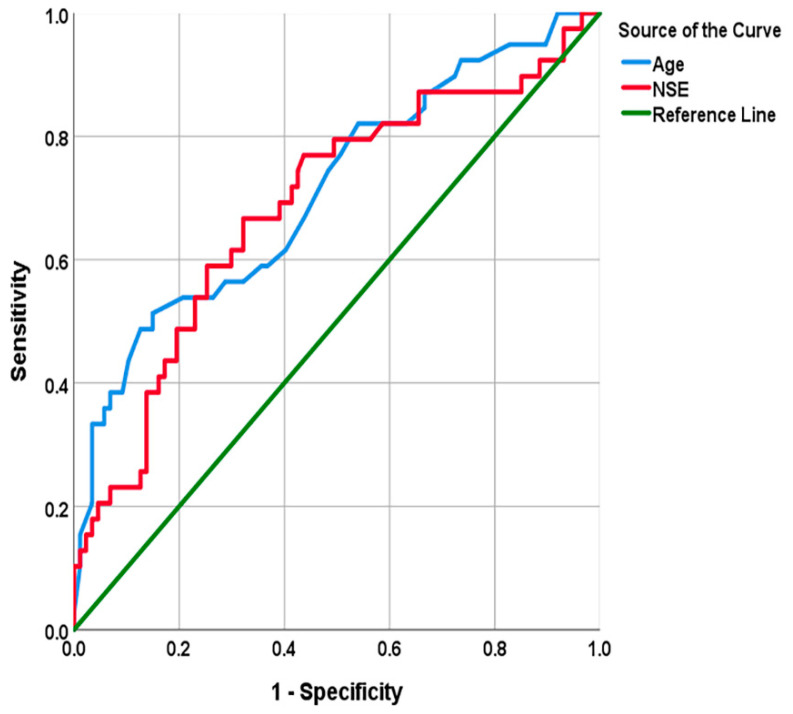
Receiver operating characteristic (ROC) plots for age and NSE.

The results of binary logistic regression analysis are summarized in Table 3. Notably, age (OR: 1.11; *p* = 0.035; 95%CI: 1.01–1.23) and serum NSE (OR: 1.25; *p* = 0.024; 95%CI: 1.03–1.51) levels were independent predictors. Therefore, elevated levels of age or NSE may be correlated with a poor prognosis in naSAH. The Hosmer–Lemeshow test shows that the goodness of fit of the logistic regression models is not good (*p* = 0.026). A ROC analysis (Table 4) was performed to assess the predictive ability of age and NSE. Table 4 shows that patients older than 69 years old (AUC: 0.71; 95%CI: 0.61–0.81) or patients with serum NSE levels higher than 13.75μg/L (AUC: 0.68; 95%CI: 0.58–0.79) tend to have a poor outcome (Figure 5).

### 3.6. The Multi-Factor Discriminant Model Can Make up for the Poor Goodness of Fit of the Logical Regression Model

**Figure 6:**
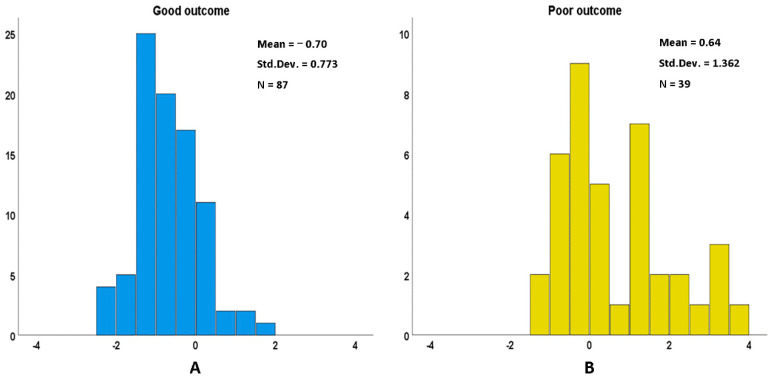
Canonical discriminant function. (**A**) Good outcome. (**B**) Poor outcome.

In fact, the logistic regression results show that several other indicators also have a discriminant effect on the prognosis, including D-dipolymer, RBG, urea and FT3, although they are not independent predictors. To make up for the lack of goodness of fit of the logistic regression model, we tried to incorporate the above variables and independent predictors into the discriminant analysis and establish a more accurate prediction model. Table 5 exhibited the results of the discriminant analysis model. Internal cross-validation showed that the prediction accuracy was 80.2%, although there was an overlap in the discriminant histogram of the good and poor prognosis groups (Figure 6).

In order to further observe the correlation between the hematological indicators and the prognosis of patients with an naSAH, 126 patients were divided into two groups according to the cut-off value of age determined by the ROC curve analysis in the present study. The results showed that there were many hematological indicators significantly correlated with prognosis in patients with an naSAH over 69 years old (Table 6). However, only D-dipolymer was significantly correlated with the prognosis in patients with an naSAH ≤ 69 years old.

## 4. Discussion

The current study investigated patients with aSAHs and naSAHs. First, we observed the differences in the demographic characteristics and clinical variables between the aSAH and naSAH groups. The results indicated that there was no significant difference in other variables except for the level of monocytes and the prevalence of diabetes between the two groups. Second, we focused on the prognosis of patients with an naSAH and tried to identify independent predictors associated with a poor prognosis. This study demonstrates that age and the level of serum NSE can predict the clinical outcome after three months independently. In addition, we developed a predictive model using discriminant analysis, which incorporated age and several hematological indicators, including D-dipolymer, NSE, RBG, urea and FT3. Last but not least, we analyzed the correlation between prognosis and hematological indicators in patients of different ages.

Whether pathological vasospasm and delayed cerebral ischemia after an angiogram-negative SAH and aneurysmal SAH are similar remains unknown [2]. Delayed cerebral ischemia (DCI) is a late complication occurring typically 4–14 days after the onset of an SAH induced by a combination of angiographic vasospasm, arterial constriction and thrombosis, as well as cortical spreading ischemia [8]. DCI is an important factor affecting about 30% of survivors and seems to be a major contributor to poor outcomes after an aSAH [9]. Gusdon et al. [8]. found that the monocyte count robustly predicted DCI and outcomes, in a sex dependent fashion. A prospective study [10] demonstrated an increased monocyte infiltration secondary to aneurysm rupture in patients with DCI. Moreover, early brain injury (EBI) is the direct brain lesion resulting from the initial bleeding in the acute stage of an SAH, which occurs within the first 3 days after an SAH and is one of the primary causes of the poor prognosis after an SAH [11,12,13]. Inflammatory responses following an SAH, an important pathological process in EBI, include the activation of resident microglia and subsequent infiltration of peripheral monocytes [14]. The present study suggests that monocytes may play a more important role in aSAHs.

An increased risk of cardiovascular disease is well recognized in patients with DM. Population-based studies involving SAH-related risk factors have shown that diabetes (both types 1 and 2) is not associated with an increased risk of SAH [15]. It is important to note, however, that most of these studies were conducted in the cohort of aSAH patients. A previous multicenter prospective cohort research [15] reported that naSAH was a distinct new microvascular complication in type 1 diabetes. Although the present data displayed that the proportion of diabetic patients in the naSAH group was higher than in the aSAH group, the study neither distinguished the subtypes of diabetes, nor observed a significant difference in RBG levels between naSAH and aSAH patients.

Therefore, it is necessary to establish a prospective cohort of different diabetes subtypes to observe the dynamic changes in fasting blood glucose, postprandial blood glucose and glycosylated hemoglobin in aSAH and naSAH patients.

In order to accelerate the turnover rate of patients, naSAH patients are often discharged for rehabilitation as soon as possible, which emphasizes the necessity of prognostic prediction in naSAH patients [7]. Age is a demographic variable, and aging is a risk factor for many diseases. Importantly, the influence of age on SAH is related to a variety of factors, such as the average age and the level of aging of the region [16]. A systematic review [16] showed that the incidence of SAH in women was 1.24 (1.09 to 1.42) times higher than in men; this gender difference started at age 55 years and increased thereafter. In fact, when the age is older than 50 years old, the probability of a poor outcome begins to enhance gradually and rises sharply after the age of 70 [17,18]. The relationship between age and poor outcomes is not linear for patients admitted in WFNS grades 1–3, who are termed good-grade patients [19]. On the other hand, poor-grade patients (WFNS grades 4 and 5) exhibited a linear increase in poor outcomes as age increased [19]. Additionally, higher age independently correlated with the occurrence of an intraventricular hemorrhage [20].

To the best of our knowledge, most studies involving the prognosis of naSAH support that clinical and radiographic scales are of great significance to the prognosis. However, there are few studies that have explored the relationship between hematological indicators and the prognosis of naSAHs.

NSE is a glycolytic enzyme in the cytoplasm of neurons that is released in the setting of cell death [21]. The NSE level is well recognized as a tool for neuroprognostication [22]. Nevertheless, the NSE level and its diagnostic and predictive ability are related to many factors. A prospective study [23] of biomarkers involving aSAH patients suggested that the difference in the cerebrospinal fluid (CSF) NSE levels between the control and the aSAH groups was statistically significant on days 5 (*p* = 0.030) and 7 (*p* = 0.039) after an SAH, whereas the serum NSE levels showed a significant difference within the first 3 days after an SAH. However, a recent retrospective research conducted by Arca et al. [21] confirmed that CSF-NSE can better reflect the volume of cerebral infarction and stroke severity in patients with neonatal arterial ischemic stroke. Another prospective study [24] found that the serum NSE (AUC = 0.83; Se = 0.69; Sp = 0.89) level on day 7 after an SAH had predictive value for the prognosis at 6 months after an SAH, and the threshold was 14.5 μg/L. In a clinical trial [25], serum NSE levels were used to evaluate the severity of an SAH before and after drug intervention. Although a large number of prospective studies have illustrated the role of NSE in aSAH, no relevant studies have been conducted in the naSAH cohort.

In the current study, several hematological indicators were analyzed. Although only NSE was an independent predictor of the prognosis of 3 months after the naSAH, we found several other hematological parameters with significant differences between poor and good outcome groups in the univariate analysis. D-dipolymer is the production of fibrin degradation [26]. D-dipolymer elevation may reflect hemostatic and coagulopathic disturbances occurring in multiple organ systems [26]. Retrospective researches [26,27] of Fukuda et al. reported that elevated D-dimer levels upon admission were not only significantly associated with an increased incidence of thromboembolic events during the endovascular coiling of ruptured aneurysms, but also independently correlated with systemic complications and the clinical outcomes of patients with an aSAH. The present study confirmed that plasma D-dipolymer levels were also elevated in naSAH patients with a poor prognosis. What is more, our study suggests that RBG has a certain discriminant effect on the prognosis of patients with an naSAH. A retrospective study [3] of Zhang et al. collected the data of admission blood glucose, fasting blood glucose and random blood glucose of patients with an naSAH, and found that stress-induced hyperglycemia was a significant and independent risk factor for symptomatic vasospasm, delayed cerebral infarction, hydrocephalus and a long-term poor outcome in naSAH patients.

In addition, binary logistic regression found that urea and FT3 levels in the blood had an impact on the prognosis of naSAHs. At present, there is no relevant research to confirm this. However, studies [28,29] concerning the prognosis of the neuroendocrine function of aSAHs suggest that SAHs may lead to pituitary dysfunction, resulting in a variety of hormone secretion disorders. We think this may cause the decrease in FT3 levels in the blood in an indirect way.

Combining the above parameters, this study established a multi-factor prediction model, and six clinical indicators (age, NSE, D-dipolymer, RBG, urea and FT3) were included in the discriminant function. This is the first time an naSAH prognostic model by discriminant analysis has been developed.

There are several limitations in the present study. First, this is a relatively small single center study and fewer patients with an SAH were included. Second, we generally collected hematological indicators of patients during hospitalization, while we ignored the dynamic changes in these variables. In addition, we only observed the outcomes of naSAH patients after three months, but seldom focused on the complications during the course of naSAH patients. Finally, this study has the inherent biases of a retrospective study, and, as an exploratory work, we did not externally validate the multivariate model during the research.

## 5. Conclusions

The blood monocyte count was higher in aSAH patients, while the proportion of DM patients was higher in naSAH patients. Increased age (>69) and serum NSE level (>13.75 μg/L) were independently correlated with a poor outcome of patients with an naSAH after three months. D-dipolymer, RBG, urea and FT3 levels are the major predictors of prognosis of patients with an naSAH, and the FT3 level decreased in patients with a poor outcome. Internal validation showed that the accuracy of the six-variable discriminant model (age, NSE, D-dipolymer, RBG, urea and FT3) for prognosis is 80.2%. Moreover, hematological indicators may have a higher prognostic value in patients over 69 years of age with an naSAH.

## Figures and Tables

**Table 1:** Comparison between aSAH and naSAH.

Test Result Variables	Non-Aneurysm	Aneurysm	*p*-Value
**Number**	126	69	
**Age**	62.02 ± 14.23	61.94 ± 14.41	0.973 ^a^
**Hospitalization days**	15.00 (9.50)	17.00 (9.00)	0.052 ^b^
**WBC**	10.76 ± 4.12	10.99 ± 3.77	0.706 ^a^
**Lymphocyte**	1.40 (1.00)	1.30 (0.90)	0.390 ^b^
**Monocyte**	0.50 ± 0.26	0.60 ± 0.27	0.003 ^a^
**Neutrophil**	8.54 ± 4.16	8.70 ± 3.90	0.798 ^a^
**NLR**	5.51 (8.71)	7.08 (7.27)	0.502 ^b^
**PT**	11.35 (1.30)	11.10 (1.30)	0.056 ^b^
**APTT**	25.04 ± 3.94	24.25 ± 3.45	0.163 ^a^
**D-dipolymer**	1.12 (1.98)	1.56 (2.80)	0.208 ^b^
**NSE**	13.02 (6.13)	13.67 (5.31)	0.082 ^b^
**RBG**	7.62 (3.25)	7.50 (3.10)	0.864 ^b^
**TC**	4.87 ± 1.20	4.72 ± 1.03	0.358 ^a^
**TG**	1.37 (0.70)	1.24 (0.75)	0.359 ^b^
**HDL**	1.26 ± 0.37	1.31 ± 0.37	0.391 ^a^
**LDL**	3.00 ± 1.06	2.91 ± 0.81	0.475 ^a^
**Homocysteine**	12.59 (6.22)	14.30 (9.01)	0.564 ^b^
**AST**	27.00 (12.50)	30.50 (8.80)	0.292 ^b^
**ALT**	30.50 (21.30)	29.00 (9.80)	0.411 ^b^
**Urea**	5.80 (2.30)	5.75 (3.80)	0.779 ^b^
**Cr**	60.00 (24.50)	64.50 (27.00)	0.680 ^b^
**UA**	305.18 ± 88.63	299.89 ± 101.72	0.797 ^a^
**FT3**	3.50 ± 0.92	3.43 ± 0.74	0.724 ^a^
**FT4**	16.02 ± 2.57	16.35 ± 2.19	0.537 ^a^
**TSH**	0.76 (0.90)	0.59 (0.98)	0.207 ^b^
**Gender**			0.220 ^c^
**Male**	70 (55.6)	32 (46.4)	
**Female**	56 (44.4)	37 (53.6)	
**Pneumonia**	45 (35.7)	25 (36.2)	0.943 ^c^
**GCS**			0.169 ^d^
**15**	59 (46.8)	23 (33.3)	
**13~14**	24 (19.0)	10 (14.5)	
**9~12**	11 (8.7)	15 (21.7)	
**3~8**	32 (25.4)	21 (30.4)	
**mFS**			0.164 ^d^
**1**	51 (40.5)	17 (24.6)	
**2**	18 (14.3)	16 (23.2)	
**3**	13 (10.3)	9 (13.0)	
**4**	44 (34.9)	27 (39.1)	
**HBP**	65 (51.6)	43 (62.3)	0.149 ^c^
**DM**	38 (30.2)	10 (14.5)	0.015 ^c^
**Smoking history**	20 (15.9)	10 (14.5)	0.798 ^c^
**Drinking history**	14 (11.1)	6 (8.7)	0.595 ^c^

Data presented as mean ± standard deviation (SD), median (interquartile range) and number (percentage). *p*-values are determined by t ^a^, Mann–Whitney U ^b^, Pearson chi-square ^c^, and Cochran–Armitage trend ^d^ tests between aSAH and naSAH groups. WBC, white blood cell count; NLR, neutrophil–lymphocyte ratio; PT, prothrombin time; APTT, activated partial thromboplastin time; RBG, random blood glucose; TC, total cholesterol; TG, triglyceride; HDL, high-density lipoprotein; LDL, low-density lipoprotein; AST, aspartate transaminase; ALT, alanine transaminase; Cr, creatinine; UA, uric acid; FT3, free triiodothyronine; FT4, free tetraiodothyronine; TSH, thyrotropin.

**Table 2:** Baseline data and post-admission data of non-aneurysm patients.

Test Result Variables	All Patients	90-Day mRS:0–2	90-Day mRS:3–6	*p*-Value
**Number**	126	87 (69.0)	39 (31.0)	
**Age**	62.02 ± 14.23	58.45 ± 12.26	70.03 ± 15.17	<0.001 ^a^
**Hospitalization days**	15.00 (9.50)	15.00 (10.00)	13.00 (15.00)	0.058 ^b^
**WBC**	10.76 ± 4.12	10.16 ± 3.37	12.11 ± 5.24	0.037 ^a^
**Lymphocyte**	1.40 (1.00)	1.30 (1.10)	1.40 (1.10)	0.696 ^b^
**Monocyte**	0.50 ± 0.26	0.46 ± 0.22	0.59 ± 0.31	0.016 ^a^
**Neutrophil**	8.54 ± 4.16	8.07 ± 3.68	9.60 ± 4.95	0.089 ^a^
**NLR**	5.51 (8.71)	5.33 (8.36)	6.41 (10.57)	0.458 ^b^
**PT**	11.35 (1.30)	11.30 (1.20)	11.40 (1.30)	0.937 ^b^
**APTT**	25.04 ± 3.94	25.23 ± 3.90	25.64 ± 4.04	0.439 ^a^
**D-dipolymer**	1.12 (1.98)	0.97 (1.11)	3.40 (7.38)	0.001 ^b^
**NSE**	13.02 (6.13)	12.18 (5.16)	15.38 (6.27)	0.001 ^b^
**RBG**	7.62 (3.25)	6.96 (2.51)	8.20 (3.58)	0.003 ^b^
**TC**	4.87 ± 1.20	4.99 ± 1.26	4.62 ± 1.02	0.117 ^a^
**TG**	1.37 (0.70)	1.41 (0.72)	1.36 (0.67)	0.409 ^b^
**HDL**	1.26 ± 0.37	1.27 ± 0.39	1.24 ± 0.34	0.764 ^a^
**LDL**	3.00 ± 1.06	3.13 ± 1.09	2.73 ± 0.96	0.055 ^a^
**Homocysteine**	12.59 (6.22)	12.56 (6.09)	14.16 (13.63)	0.293 ^b^
**AST**	27.00 (12.50)	24.50 (12.80)	32.50 (19.00)	0.019 ^b^
**ALT**	30.50 (21.30)	31.50 (20.00)	28.50 (26.80)	0.830 ^b^
**Urea**	5.80 (2.30)	5.70 (2.10)	7.40 (3.30)	0.006 ^b^
**Cr**	60.00 (24.50)	59.50 (24.80)	63.50 (33.00)	0.122 ^b^
**UA**	305.18 ± 88.63	296.85 ± 87.68	340.86 ± 86.78	0.095 ^a^
**FT3**	3.50 ± 0.92	3.68 ± 0.81	2.84 ± 1.03	0.001 ^a^
**FT4**	16.02 ± 2.57	15.94 ± 2.42	16.30 ± 3.14	0.622 ^a^
**TSH**	0.76 (0.90)	0.82 (0.94)	0.75 (0.58)	0.331 ^b^
**Gender**				0.518 ^c^
**Male**	70 (55.6)	50 (57.5)	20 (51.3)	
**Female**	56 (44.4)	37 (42.5)	19 (48.7)	
**Pneumonia**	45 (35.7)	24 (27.6)	21 (53.8)	0.004 ^c^
**GCS**				<0.001 ^d^
**15**	59 (46.8)	56 (64.4)	3 (7.7)	
**13~14**	24 (19.0)	19 (21.8)	5 (12.8)	
**9~12**	11 (8.7)	5 (5.7)	6 (15.4)	
**3~8**	32 (25.4)	7 (8.0)	25 (64.1)	
**mFS**				<0.001 ^d^
**1**	51 (40.5)	45 (51.7)	6 (15.4)	
**2**	18 (14.3)	15 (17.2)	3 (7.7)	
**3**	13 (10.3)	9 (10.3)	4 (10.3)	
**4**	44 (34.9)	18 (20.7)	26 (66.7)	
**HBP**	65 (51.6)	38 (43.7)	27 (69.2)	0.008 ^c^
**DM**	38 (30.2)	21 (24.1)	17 (43.6)	0.028 ^c^
**Smoking history**	20 (15.9)	16 (18.4)	4 (10.3)	0.248 ^c^
**Drinking history**	14 (11.1)	12 (13.8)	2 (5.1)	0.261 ^e^

Data presented as mean ± standard deviation (SD), median (interquartile range) and number (percentage). *p*-values are determined by t ^a^, Mann–Whitney U ^b^, Pearson chi-square ^c^, and Cochran–Armitage trend ^d^, likelihood ratio chi-square ^e^ tests between good and poor outcome.

**Table 3:** Binary logistic regression analysis.

Test Result Variables	Coefficient	OR	95%CI for OR	*p*-Value
**Age**	0.11	1.11	[1.01, 1.23]	0.035
**D-dipolymer**	0.21	1.23	[0.96, 1.58]	0.098
**NSE**	0.22	1.25	[1.03, 1.51]	0.024
**FT3**	−1.34	0.26	[0.06, 1.10]	0.067
**Constant**	−9.02			0.069

**Table 4:** Receiver operator characteristic (ROC) analysis.

Test Result Variables	AUC	95%CI	*p*-Value	Cut-Off Value	Se	Sp	J
**Age**	0.71	[0.61, 0.81]	0.000	69.00	0.51	0.85	0.36
**NSE**	0.68	[0.58, 0.79]	0.001	13.75	0.67	0.68	0.35

Se, sensitivity; Sp, specificity; J, Youden index.

**Table 5:** Canonical discriminant function coefficients.

Parameters	Coefficients	Canonical Correlation	*p*-Value
**Age**	0.028	0.775	<0.001
**D-dipolymer**	0.143		
**NSE**	0.082		
**RBG**	0.054		
**Urea**	0.115		
**FT3**	−0.466		
**(Constant)**	−3.157		

**Table 6:** Correlation analysis of hematological indicators and mRS in different age groups.

Test Result Variables	All Patients (N = 126)		Age ≤ 69 Years (N = 93)		Age > 69 Years (N = 33)	
	R	*p*-Value	R	*p*-Value	R	*p*-Value
**D-dipolymer**	0.4	0.001	0.3	0.018	0.3	0.267
**NSE**	0.3	0.003	0.2	0.078	0.3	0.078
**RBG**	0.3	0.001	0.2	0.109	0.6	<0.001
**Urea**	0.3	0.008	0	0.820	0.6	0.004
**FT3**	−0.4	0.001	−0.2	0.179	−0.5	0.026

R, Spearman’s rank correlation coefficient.

## Data Availability

The data that support the findings of this study are available from the corresponding author upon reasonable request.
